# Predicting criminal offence in adolescents who exhibit antisocial behaviour: a machine learning study using data from a large randomised controlled trial of multisystemic therapy

**DOI:** 10.1007/s00787-024-02592-7

**Published:** 2024-10-08

**Authors:** Jae Won Suh, Rob Saunders, Elizabeth Simes, Henry Delamain, Stephen Butler, David Cottrell, Abdullah Kraam, Stephen Scott, Ian M Goodyer, James Wason, Stephen Pilling, Peter Fonagy

**Affiliations:** 1 CORE Data Lab, Centre for Outcomes Research and Effectiveness, Research Department of Clinical, Educational and Health Psychology, University College London, London, UK; 2 Research Department of Clinical, Educational and Health Psychology, University College London, London, UK; 3 Department of Psychology, University of Prince Edward Island, Charlottetown, Canada; 4 Leeds Institute of Health Sciences, University of Leeds, Leeds, UK; 5 Institute of Psychiatry, Psychology and Neuroscience, National Academy for Parenting Research, Kings’s College London, London, UK; 6 Department of Psychiatry, University of Cambridge, Cambridge, UK; 7 Population Health Sciences Institute, Newcastle University, Newcastle upon Tyne, UK; 8 University of Leeds, Leeds, UK

**Keywords:** Criminal offending, Recidivism, Youth crime, Machine learning, Prediction modelling

## Abstract

**Introduction:**

Accurate prediction of short-term offending in young people exhibiting antisocial behaviour could support targeted interventions. Here we develop a set of machine learning (ML) models that predict offending status with good accuracy; furthermore, we show interpretable ML analyses can complement models to inform clinical decision-making.

**Methods:**

This study included 679 individuals aged 11–17 years who displayed moderate-to-severe antisocial behaviour, from a controlled trial of Multisystemic-therapy in England. The outcome was any criminal offence in the 18 months after study baseline. Four types of ML algorithms were trained: logistic regression, elastic net regression, random forest, and gradient boosting machine (GBM). Prediction models were developed (1) using predictors readily available to clinicians (e.g. sociodemographics, previous convictions), and (2) with additional information (e.g. parenting). Model agnostic feature importance values were calculated and the most important predictors identified. Nested cross-validation with 100 iterations of random data splits and 10-fold cross-validation within each iteration was employed, and the average predictive performance was reported.

**Results:**

Among the ML models using readily available predictors, the GBM is the strongest model (AUC 0.85, 95% CI 0.85–0.86); the other models have average AUCs of 0.82. This performance was better than using only the total number of previous offences as the predictor (0.67, 0.66–0.68), and the model simply assuming past offending status as the prediction (0.81, 0.80–0.81). Additional predictors slightly increased the performance of logistic regression and random forest models but decreased the performance of elastic net regression and gradient boosting machine-based models.

**Conclusion:**

The potential utility of ML approaches for accurately predicting criminal offences in high-risk youth is demonstrated. Interpretable ML-based predictive models could be utilised in youth services or research to help develop and deliver effective interventions.

**Supplementary Information:**

The online version contains supplementary material available at 10.1007/s00787-024-02592-7.

## Introduction

Among young individuals, conduct disorders and related antisocial behaviour are the predominant mental and behavioural issues [1, 2]. Conduct disorder affects approximately 3.6% of those between the ages of 10–14 and 2.4% of 15–19 year olds, as estimated by the World Health Organization (WHO) [3]. Young individuals who demonstrate antisocial behaviour often participate in criminal activities, which detrimentally impact their long-term mental and physical health, job prospects, social networks, and society as a whole [1, 4–6].

Early-life interventions, like social skills training and educational support for children displaying disruptive behaviour in school alongside parent training, have been developed with the aim of preventing future criminal behaviour [7]. A systematic review and meta-analysis of childhood interventions indicated that their benefits were largely moderate and inconsistent across different studies [8]. However, these interventions were generally offered to low-income families or to children deemed ‘high-risk’ based on teachers’ subjective assessments. Therefore, individuals who were truly at the highest risk of offending may not have been included in these trials.

To deliver intensive interventions when and where they are most needed, it is crucial to be able to accurately predict short-term offending in young individuals who demonstrate antisocial behaviour. Several potential predictors have been indicated in evaluating whether a child displaying antisocial behaviour is at increased risk of offending, and thus in need of suitable support. These include characteristics that are likely to be available for those who provide health and social care, including age, gender, socioeconomic status, and previous convictions [9–12]. Previous offending alone is a strong predictor of future offending [5, 13, 14], but additional characteristics, such as child and parent mental health, family functioning, and educational participation (which may be less accessible to most care professionals) may enhance clinical assessments and practice if they can refine predictions of later offending.

Previous studies in the wider psychological literature have leveraged various methodologies for predicting offending and recidivism. One class of methods that stands out for its efficacy in handling large and intricate data is machine learning (ML) [14]. Although a few studies have utilised ML for predicting adolescent crime linked to antisocial behaviour, these models frequently did not outperform logistic regression models [14, 15]. Some reasons for the relatively poor performance of existing models may include the small sample size, limited selection of algorithms and features tested, and suboptimal hyperparameter tuning [16, 17]. For instance, one study demonstrated that a widely used reoffending risk prediction software including up to 137 predictors was no more accurate than a logistic regression model with merely two predictors (age and total number of previous convictions) [13]. Another challenge is that ML models can be ‘black boxes’, where their operation and basis are unclear, posing difficulties for interpretation and application in routine clinical settings [18]. Despite these hurdles, ML models are well-poised to learn complex relationships from numerous predictor variables; this is typically beyond the reach of traditional statistical methods [19]. Furthermore, recent developments in the field of interpretable ML have increased confidence that models can be better understood.

The creation of an accurate and interpretable prediction model of criminal behaviour is critical for several reasons. First, it allows for the implementation of early intervention strategies, aimed at mitigating risk factors and strengthening protective factors. Effectively, this can prevent the onset of criminal behaviour. In addition, models can help identify individuals that will most likely benefit from interventions, leading to more efficient allocation of resources within the criminal justice and social support systems. Finally, an accurate and interpretable model provides a basis for shaping policies and practices to better manage potential risks, contributing to safer communities.

In this work, we develop robust ML-based prediction models for criminal offending among young individuals displaying antisocial behaviours. We leverage a large dataset comprised of 679 individuals with antisocial behaviour and up to 65 features using four different ML algorithms, and compare our approach to traditional statistical models. Finally, we underscore the validity of our models by employing interpretable ML techniques to elucidate the features that most significantly influence predictions.

## Methods

This study followed the Enhancing the Quality and Transparency of Health Research (EQUATOR) reporting guideline: Transparent reporting of a multivariable prediction model for individual prognosis or diagnosis (TRIPOD) [20] (Supplement E).

### Data source

The data utilised for analysis in this study was drawn from the Systemic Therapy for At Risk Teens (START) study, a pragmatic, randomised-controlled, superiority trial conducted at nine multisystemic therapy pilot centres in England from February 4, 2010, to September 1, 2012. Previous publications have provided a detailed report on the design and findings of the START trial [21, 22]. In brief, the study encompassed:

1. Population: participants aged 11–17 years with moderate-to-severe antisocial behaviour who had at least three severity criteria indicating past difficulties across several settings and one of five general inclusion criteria for antisocial behaviour.
2. Intervention: 3–5 months of multisystemic therapy (MST) followed by management as usual (*n* = 342).
3. Comparison: management as usual (*n* = 342).
4. Primary outcome: out-of-home placement at 18 months (the target sample size for the trial was calculated to have 86% power to detect a 20% reduction in out-of-home placement).

Secondary outcomes encompassed time to first criminal offence, the total number of offences, and a variety of measures of antisocial behaviour and attitudes, assessed by both parents and the young participants. At the 18-month mark, there was no statistically significant difference between groups in the proportion of participants in out-of-home placements. Further, there were no long-term benefits concerning behaviour, mental health, social care, forensics, or education, nor any economic advantage, for MST compared with usual management.

### Outcome

The outcome intended to be predicted in this study was the occurrence of any criminal offence during the entire 18-month follow-up period from the study baseline (i.e., post-randomisation in the START trial). Data on criminal offences was sourced from the official records of the Police National Computer and Young Offender Information System. (This was an important secondary outcome in the START trial.)

### Predictors

All potential predictors accessible for training the prediction models were measured at the study baseline. These included sociodemographic characteristics, questionnaire measures of antisocial behaviour and attitudes, well-being and adjustment, psychiatric disorders, parenting skills, and participation in education of both the young participants and their parents, as applicable. Predictors were classified into two categories: the ‘minimal’ predictors, which are predictors readily accessible to decision-makers providing health and social care to young people with antisocial behaviour, and the ‘additional’ predictors, which are available for the START study, but not necessarily in routine clinical settings. The list of minimal predictors is presented in Table 1, and the categories of the minimal predictors and list of additional predictors are available in Supplement A. Further details on the data collection for each predictor can be found in the initial publication of the START study [21].

**Table 1:** List of predictors that are readily available to care providers

Minimal predictors (readily available to care providers and decision-makers)	Description and categories	Data source
**Referral and intervention**		
Site	Refers to the region where recruitment to the trial took place. Categories: Barnsley, Greenwich, Hackney, Leeds, Merton, Peterborough, Reading, Sheffield, and Trafford.	START Trial
Source of referral	Indicates how each young person was referred to the trial. Categories: Social Services, Youth Offending Teams, Education Services, Child and Adolescent Mental Health Services (CAMHS), Police Triage and Other (including Housing Services). All young people were first referred to local multi-agency panels to standardise the referral process; these panels identified the suitability of multisystemic therapy for each participant and invited them for formal assessment for the trial.	START Trial
Intervention assigned in the START trial	Categories: Multisystemic therapy (MST) or Management as usual (MAU). MST is an intensive family and home-based intervention for young people with serious antisocial behaviour, which aims to prevent reoffending and out-of-home placements [23]. MAU was provided to all families by youth offending teams, CAMHS, or social and education services as needed, in line with national treatment guidelines [24, 25]. Interventions were individualised to the young person’s mental health needs and behavioural difficulties.	START Trial
**Offences in year before referral**		
Offender on referral	Whether or not the young person had a record of offence in the year prior to referral.	Police National Computer database and Young Offending Information System
Total number of offences	Total number of offences in the year prior to referral.	Police National Computer database and Young Offending Information System
**Demographic and background information**		
Age	Age of the young person (years).	Family information form
Gender	Gender of the young person (Male or Female).	Family information form
Ethnicity	Ethnicity of the young person (White or Non-White).	Family information form
Socioeconomic status	Based on total household income before tax. Categories: Low (Less than £10,000), Medium (£10,001-£30,000), and High (£31,000 or above).	Family information form
Number of siblings	Number of siblings of the young person.	Family information form
Parents’ marital status	Categories: ‘Married or co-habiting’ or ‘Not married or co-habiting’ (including single, widowed, separated, or divorced).	Family information form
Parents’ highest educational qualification	‘No qualifications’ or ‘Any qualification’ (O levels and above)	Family information form
Parents’ employment status	Unemployed or ‘Employed or homemaker’	Family information form
Other children offended	Whether any one of the parent’s other children (not enrolled on the trial) has been involved in offending behaviour.	Family information form
Parent offended	Whether the parent has ever been involved as an adult in offending behaviour.	Family information form
IQ	IQ estimates were obtained for youths using the WASI II, an IQ test suitable for administration from ages 6 and up, including an evaluation of general intelligence as well as verbal and performance intelligence.	Wechsler Abbreviated Scale of Intelligence (WASI II)
Young person accommodation	Categories: ‘Living at home’ or ‘Not living at home’. From CA-SUS, a questionnaire developed specifically for the trial, designed to record all contact with health, social care, and criminal justice services. Completed by both the parent/caregiver and young person.	Child and adolescent service use schedule (CA-SUS)
**Comorbid psychiatric diagnosis**		
Conduct disorder	The young person’s psychiatric disorders were identified by the DAWBA [26], a computerised structured interview measure.	Development and Well-Being Assessment (DAWBA)
Attention Deficit Hyperactivity Disorder		Development and Well-Being Assessment (DAWBA)
Depression		Development and Well-Being Assessment (DAWBA)

### Statistical analysis

The study sample consisted of 679 participants, with four individuals excluded due to missing information regarding the outcome (offending according to police records).

Four supervised ML algorithms (logistic regression, elastic net regression [27], random forest [28], and gradient boosting machine [29]) were employed to train classification models to predict criminal offence in police records over the subsequent 18 months. These algorithms were chosen due to their common use in prior studies and their capacity to retrospectively identify features that are crucial for predicting new data [14]. Further details on each ML algorithm are provided in Supplement B. The ML models were contrasted with two null models: (1) a logistic regression model that utilised only the number of offences recorded in the year prior to the study baseline as a single predictor, and (2) using offending status at the study baseline exactly as the prediction (i.e., presuming that all baseline offenders will re-offend, and that all baseline non-offenders will remain offence-free).

ML models were first trained using the minimal predictors. These predictors were derived from routinely-collected data or information that care providers can easily measure, such as sociodemographic characteristics and previous criminal offence records (see Table 1). The optimal combination of hyperparameters for each algorithm, excluding logistic regression, was identified by grid searching on the training set (see Supplement C for details). These algorithms are available as saved Python classes [30], and can be replicated using the hyperparameter settings presented in Supplement C. The models are trained via the ‘fit’ method and used to generate individual predictions via the ‘predict’ method. As a further analysis, models were trained using all 65 available predictors to ascertain whether additional predictors might enhance predictive performance. These predictors, detailed in Supplementary Table A2, encompassed various measures of antisocial behaviour and attitudes in the young person and their parents, mental health and well-being of the young person and parents, parenting skills, family functioning, and educational participation.

In order to estimate the variance in performance that can arise from how we partition the training and test sets, and to separate data pre-processing and hyperparameter optimisation from the final model validation, we implemented nested cross-validation [31]. This includes an outer and an inner validation loop. In each iteration of the outer loop, the full dataset was divided into training data (80%) and testing data (20%), stratified by the outcome. Missing values in the predictors were imputed separately for the training and testing datasets after the split, using a nonparametric imputation method for mixed-type data via the Python implementation of the ‘missForest’ package in R [32]. In the inner validation loop, we trained the models on the imputed training dataset via 10-fold cross-validation, and obtained performance metrics from the imputed test dataset. We then averaged the performance metrics for each type of model over 100 iterations of the outer loop. In other words, we repeated the entire modelling pipeline 100 times, each with a different random seed, which resulted in a distinct subset of 80% of participants forming a new training set for each iteration.

The area under the receiver operating characteristic curve (AUC) in the test set served as the primary measure of model performance. The AUC is a measure of a model’s capacity to distinguish between young people who commit an offence and those who do not. Sensitivity, specificity, positive predictive value (PPV), and negative predictive value (NPV) were also evaluated as secondary model performance metrics. Expressed in terms of the elements of a confusion matrix, sensitivity is defined as True Positives/(True Positives + False Negatives). Specificity is defined as True Negatives/(True Negatives + False Positives). PPV is defined as True Positives/(True Positives + False Positives). NPV is defined as True Negatives/(True Negatives + False Negatives). Variable importance was calculated using SHAP (SHapley Additive exPlanations) [33], a model-agnostic metric that can indicate each feature’s contribution to the model’s prediction at both the individual observation and global level, thereby enhancing the models’ interpretability. We averaged the SHAP values for each predictor variables across the 100 iterations of the test sample.

## Results

### Participant characteristics

The 679 participants, aged 11–17 years (mean 13.8, SD 1.4) at baseline, included 430 (63%) males and 249 (37%) females. Among these young individuals, 292 (43%) had a police record of offending in the total 18 months since the baseline, while 387 (57%) did not. Table 2 reports the distribution of the outcome to be predicted, which shows a decrease in the number and proportion of participants with a police record of offending over the follow-up period.

**Table 2:** Distribution of the number of offences recorded during different periods of follow-up since study baseline

	Follow-up period		
Number of offences	0–6 months	6–12 months	12–18 months
0	497	520	559
1	82	78	64
2	50	30	26
3	19	20	13
4+	31	31	17

### Prediction model development and validation

Prediction models were first developed using features that are readily accessible to clinical decision-makers (see Minimal predictors, Table 1). Table 3 presents the average model performance metrics for the best performing models (i.e., with optimised hyperparameters) over 100 different random splits of the dataset. On average, all ML models predict the outcome more effectively than the single predictor logistic regression model (mean AUC 0.667, 95% CI 0.660–0.675), which used only the total number of offences in the year prior to study baseline as a predictor.

The performance of multivariable logistic regression (mean AUC 0.822, 95% CI 0.816–0.828), elastic net regression (0.819, 0.813–0.825), and random forest models (0.823, 0.817–0.830) are somewhat better than assuming that offender or non-offender status at baseline perfectly forecasts future offending behaviour (0.807, 0.803–0.812). However, this null model has artificially high sensitivity and NPV due to none of the non-offenders at baseline in the dataset having a record of offending during follow-up, and its specificity is markedly lower than the multivariable logistic regression, elastic net regression, and random forest models. The gradient boosting machine has the highest performance in our test datasets (0.853, 0.848–0.859).

We find that adding more features improves the average AUCs of the multivariable logistic regression and random forest. Adding more features does not benefit the elastic net regression or gradient boosting machine (Table 3), but the gradient boosting machine remains the best performing algorithm (mean AUC 0.848, 95% CI 0.842–0.854).

**Table 3:** Average model performance over 100 random test sets

Minimal model	AUC	Sensitivity (Recall)	Specificity	PPV (Precision)	NPV
**Average performance**		**Mean (95% CI)**			
Multivariable logistic regression	0.822 (0.816;0.828)	0.852 (0.841;0.862)	0.685 (0.676;0.694)	0.669 (0.662;0.675)	0.863 (0.854;0.872)
Elastic net regression	0.819 (0.813;0.825)	0.826 (0.814;0.838)	0.685 (0.675;0.695)	0.662 (0.655;0.669)	0.844 (0.835;0.852)
Random forest	0.823 (0.817;0.830)	0.825 (0.813;0.836)	0.701 (0.691;0.711)	0.674 (0.666;0.682)	0.845 (0.836;0.854)
Gradient boosting machine	0.853 (0.848;0.859)	0.900 (0.883;0.917)	0.669 (0.656;0.681)	0.671 (0.664;0.678)	0.909 (0.896;0.923)
**+ additional predictors**	**AUC**	**Sensitivity (Recall)**	**Specificity**	**PPV (Precision)**	**NPV**
**Average performance**		**Mean (95% CI)**			
Multivariable logistic regression	0.832 (0.825;0.839)	0.745 (0.731;0.759)	0.741 (0.732;0.750)	0.682 (0.674;0.691)	0.798 (0.789;0.807)
Elastic net regression	0.774 (0.766;0.782)	0.587 (0.571;0.602)	0.762 (0.752;0.771)	0.648 (0.638;0.658)	0.714 (0.707;0.722)
Random forest	0.839 (0.833;0.845)	0.832 (0.820;0.844)	0.718 (0.708;0.728)	0.689 (0.682;0.696)	0.854 (0.846;0.863)
Gradient boosting machine	0.848 (0.842;0.854)	0.897 (0.881;0.913)	0.668 (0.656;0.680)	0.670 (0.663;0.677)	0.905 (0.891;0.918)
**Null models**	**AUC**	**Sensitivity (Recall)**	**Specificity**	**PPV (Precision)**	**NPV**
		**Mean (95% CI)**			
Single predictor logistic regression	0.667 (0.660;0.675)	0.364 (0.353;0.376)	0.859 (0.852;0.865)	0.658 (0.644;0.672)	0.645 (0.641;0.650)
Offender status at baseline	0.807 (0.803;0.812)	1.000 (1.000;1.000)	0.615 (0.606;0.624)	0.660 (0.655;0.665)	1.000 (1.000;1.000)

ML = Machine learning, AUC = area under the receiver operating characteristic curve, PPV = positive predictive value, NPV = negative predictive value

### Identifying the most important predictors

The five most consequential predictors for the minimal models are reported in Supplement D. Offender status at baseline is a crucial predictor across all types of ML-based models. The average feature importance values for the gradient boosting model are presented in Table 4. The paramount predictor is whether a participant had an offending record prior to the study, followed by the total number of offences in the preceding year, site (indicating the clinical trial site), IQ, and age.

**Table 4:** Average variable importance values of the top five features in the gradient boosting machine-based prediction model across the 100 random test sets

Minimal model			
Feature	Mean (|SHAP value|)	95% Confidence Interval	
Offender on referral	1.56	1.41	1.70
Total number of offences	0.27	0.24	0.30
Site of clinical trial*	0.11	0.08	0.14
IQ	0.10	0.08	0.12
Age	0.08	0.07	0.10
**+ additional predictors**			
**Feature**	**Mean (|SHAP value|)**	**95% Confidence Interval**	
Offender on referral	1.38	1.25	1.51
Total number of offences	0.15	0.13	0.17
SRD - Volume of delinquency excluding violence towards siblings	0.11	0.09	0.12
Parent-reported - Peer relationship problems score	0.07	0.06	0.08
Antisocial behaviour and attitudes	0.07	0.06	0.08

*Multi-categorical predictor variables (i.e., site of clinical trial, socioeconomic status, and source of referral) were dummy coded in the prediction models, and the variable importance metric displayed is a combination of the contribution of each of the categories.; SRD = Self-reported delinquency

Supplement D also presents variable importance values of the five most significant predictors in ML models including all additional predictors. Like in the minimal model, offender status at baseline is the most vital predictor. For multivariable logistic regression, incorporating information on self-reported delinquency appears to enhance the models’ predictive performance over the minimal model.

## Discussion

This study has developed and validated accurate ML models for predicting criminal offences in adolescents displaying antisocial behaviour. We also report the features that have the strongest attributions for predicted outcomes. On average, the gradient boosting machine is the most performant, regardless of using either the minimal or additional predictors.

This study underscores the utility of ML approaches for accurately predicting criminal offences in young people. In comparison to the null models (which only used the number of previous offences or the offender status at baseline), the ML models showed superior performance overall. Our findings, therefore, diverge from prior studies that did not identify advantages of using ML methods over simple statistical models [13, 15]. Furthermore, our models significantly outperformed existing ML-based models. A systematic review of 12 ML-based prediction models for recidivism reported an average AUC of 0.74 (range 0.69–0.78) [14], which is considerably lower than the mean AUC of 0.853 (95% CI 0.848–0.859) achieved by our gradient boosting models (using only the minimal predictors). The reasons for such differences in findings could potentially be attributed to the relatively small sample sizes and limited selection of algorithms tested in prior studies, as well as differences in data sources, outcome definitions, participant characteristics, and lack of hyperparameter optimisation.

A difference in AUC of 5% points (comparing simply assuming offenders will re-offend [mean AUC 0.807, 95% CI 0.803–0.812] versus the gradient boosting machine [0.853, 0.848–0.859]) may appear small, but at scale, these effects can be substantial. For example, correctly identifying just one additional offender at baseline who is unlikely to re-offend per 140 young people with conduct disorder could, across England, result in correctly identifying over a thousand individuals. This means that limited resources can be more efficiently re-allocated to those who are most in need. Nevertheless, it is crucial to ensure that the usage of prediction models does not inadvertently exclude those who could benefit from intervention.

The most crucial predictor across all our models was whether the young person had been an offender at the study baseline. This is consistent with the existing wealth of evidence that past crime is strongly associated with future crime, and confirms the plausibility of the main operational basis of our ML models [34–36]. The five most important predictors on average in the most performant gradient boosting models were offender status at referral, number of past offences, the site of the clinical trial, IQ, and age. All of these factors, except the site of the clinical trial, have been associated with offending and recidivism [35, 37–40]. This highlights that our ML models, far from being enigmatic ‘black boxes’, align with the established understanding of behavioural patterns in criminology.

The significance of the clinical trial site as a predictor may be due to its encapsulation of various factors, including regional socioeconomic deprivation and demographic makeup, different proportions of referral pathways for trial participants, variations in clinical practices across the sites, and their interactions. For instance, some referral pathways, such as Police Triage, may be more significant predictors of offending than others, and if these co-occur in a relatively deprived region with less clinical resources, a particular site may disproportionately influence model predictions. Moreover, if the data are clustered by site, this could partly explain the better performance of ML algorithms that account for interaction effects and can capture complex, multi-level data structure. However, it is important to note that SHAP values represent the relative importance of features in the prediction model and do not account for the quality of the predictions. The relative importance of each predictor can vary substantially depending on the specific model, and a high SHAP value does not suggest a causal relationship between the predictor and the outcome. The purpose of assessing variable importance should therefore be limited to assisting model interpretability, rather than forming the basis of causal conclusions.

The inclusion of all available predictors in this study did not always lead to improvements in predictive performance over the minimal model. While adding additional predictors improved the performance of multivariable logistic regression and random forest on average, it reduced the performance of the elastic net regression and the gradient boosting machine. This could be partly due to the issue of overfitting, a common problem in gradient boosting due to its high complexity and the interactions between many features [41, 42]. Future studies may consider refining feature selection to further improve model performance [43]. However, given the strong performance of the gradient boosting model developed using only readily available features, it is likely that there will be marginal performance gains.

### Limitations

The outcome we predicted was based solely on police records, which may not accurately reflect criminal activity that occurred prior to the collection of baseline data. Also, this method will not capture undetected crimes. Therefore, we can only draw conclusions about the model’s ability to predict criminal behaviour recorded during follow-up, rather than the actual incidence of criminal activity during this period. Furthermore, our model does not differentiate between types of offences, which can be quite diverse.

Another limitation is the need for further validation. While our tool has been developed using a sample from across England, our model has not been validated in truly independent data, and further validation is needed in more geographically and demographically diverse samples. This will help extend our findings to other countries and regions. Lastly, while we used the AUC as the main performance metric in this study, optimising for different metrics, such as sensitivity, may be more relevant for specific clinical or research purposes.

### Implications

A prediction tool capable of accurately identifying individuals likely to offend can help in preventing crime, including recidivism. Recognising potential offenders and non-offenders can allow for more precise targeting of interventions. Our study demonstrated that ML can achieve accurate predictions with just a handful of features that are easily accessible in standard clinical settings. Several features enhanced the prediction of future offending beyond simply considering past offending status, underscoring the advantages of these modelling techniques. Prediction tools like the one developed in our study could be integrated into youth services or employed in research to deliver effective interventions. The overarching objective is to intervene early to divert individuals from criminal behaviour, benefitting both the individual and society. ML holds ample potential for enabling such targeted interventions.

## Electronic supplementary material

Below is the link to the electronic supplementary material.

Supplementary Material 1

## Data Availability

Requests for access to the START data are to be made to the START research team. Analysis code is available from: https://github.com/jae-suh74/startml.
