# Supplementary material for: Predicting criminal offence in adolescents who exhibit antisocial behaviour: a machine learning study using data from a large randomised controlled trial of multisystemic therapy
Source: Eur Child Adolesc Psychiatry. 2024 Oct 8;34(5):1579–88. doi: 10.1007/s00787-024-02592-7 (PMC12122556; doi:10.1007/s00787-024-02592-7)
Supplement: Supplementary file 1 — Supplementary Material 1 [file 787_2024_2592_MOESM1_ESM.docx]

Supplementary information to:

Predicting criminal offence in adolescents who exhibit antisocial behaviour: a machine learning study using data from a large randomised controlled trial of multisystemic therapy

Jae Won Suh^1^, Rob Saunders^1^, Elizabeth Simes^2^, Henry Delamain^1^, Stephen Butler^2,3^, David Cottrell^4^, Abdullah Kraam^8^, Stephen Scott^5^, Ian M Goodyer^6^, James Wason^7^, Stephen Pilling^1^ & Peter Fonagy^2^

1. CORE Data Lab, Centre for Outcomes Research and Effectiveness, Research Department of Clinical, Educational and Health Psychology, University College London, London, UK.
2. Research Department of Clinical, Educational and Health Psychology, University College London, London, UK.
3. Department of Psychology, University of Prince Edward Island, Charlottetown, Canada.
4. Leeds Institute of Health Sciences, University of Leeds, Leeds, UK.
5. National Academy for Parenting Research, Institute of Psychiatry, Psychology and Neuroscience, Kings’s College London, London, UK.
6. Department of Psychiatry, University of Cambridge, Cambridge, UK.
7. Population Health Sciences Institute, Newcastle University, Newcastle upon Tyne, UK
8. University of Leeds, Leeds, UK

Correspondence to: Dr Jae Won Suh

Email: [j.suh@ucl.ac.uk](mailto:j.suh@ucl.ac.uk)

Journal name: European Child & Adolescent Psychiatry

Contents

[A) Further information on predictor variables 2](#_Toc169013981)

[B) Further information on machine learning algorithms 7](#_Toc169013982)

[C) Hyperparameter optimisation using grid search 8](#_Toc169013983)

[D) Top five features in machine learning-based prediction models 9](#_Toc169013984)

[E) Completed TRIPOD Checklist 10](#_Toc169013985)

# Further information on predictor variables

Table A1. Categories and missingness of minimal predictor variables

|  |  | **Total (N=679)** |
| --- | --- | --- |
| **Predictor variable** | **Categories** | **N (%)** |
| Site | Barnsley | 80 (12%) |
|  | Greenwich | 79 (12%) |
|  | Hackney | 69 (10%) |
|  | Leeds | 83 (12%) |
|  | Merton | 79 (12%) |
|  | Peterborough | 80 (12%) |
|  | Reading | 70 (10%) |
|  | Sheffield | 70 (10%) |
|  | Trafford | 69 (10%) |
| Source of Referral | Social Care | 295 (43%) |
|  | Youth Offending Service (YOS) | 117 (17%) |
|  | Child and adolescent mental health services (CAMHS) | 108 (16%) |
|  | Education | 107 (16%) |
|  | Police Triage | 12 ( 2%) |
|  | Other | 40 ( 6%) |
| Intervention assigned in the START trial | Multisystemic therapy | 340 (50%) |
|  | Management as usual | 339 (50%) |
| Offender on referral | Offender | 444 (65%) |
|  | Non-Offender | 235 (35%) |
| Gender | Female | 249 (37%) |
|  | Male | 430 (63%) |
| Ethnicity | White | 534 (79%) |
|  | Non-white | 135 (20%) |
|  | Missing | 10 ( 1%) |
| Socioeconomic status | Low | 422 (62%) |
|  | Medium | 178 (26%) |
|  | High | 66 (10%) |
|  | Missing | 13 ( 2%) |
| Parents’ marital status | Not married or co-habiting | 406 (60%) |
|  | Married or co-habiting | 269 (40%) |
|  | Missing | 4 ( 0%) |
| Parents’ educational qualification | No qualifications | 280 (41%) |
|  | Any qualification | 396 (58%) |
|  | Missing | 3 ( 0%) |
| Parents employment status | Unemployed | 160 (24%) |
|  | Employed or homemaker | 507 (75%) |
|  | Missing | 12 ( 2%) |
| Other children offended | No | 392 (58%) |
|  | Yes | 244 (36%) |
|  | Missing | 43 ( 6%) |
| Parent offended | No | 487 (72%) |
|  | Yes | 180 (27%) |
|  | Missing | 12 ( 2%) |
| Young person Accommodation | Living at home | 654 (96%) |
|  | Not living at home | 24 ( 4%) |
|  | Missing | 1 ( 0%) |
| DAWBA Conduct disorder diagnosis | No | 148 (22%) |
|  | Yes | 531 (78%) |
| DAWBA ADHD diagnosis | No | 476 (70%) |
|  | Yes | 203 (30%) |
| DAWBA depression diagnosis | No | 607 (89%) |
|  | Yes | 72 (11%) |

DAWBA=Development and Well-being Assessment

| Table A2. List of additional predictors |  |  |
| --- | --- | --- |
| **Additional predictors** |  | **Data source** |
| **Antisocial behaviour and attitudes** |  |  |
| Child-reported SDQ - Impact score | The SDQ [1] is a brief, self-reported screening instrument for assessing emotional and behavioural problems. It is used to assess and test for between-group differences regarding the severity of behavioural problems endorsed by the informant. Both the young people and their parents/caregivers completed the SDQ. | Strengths and Difficulties Questionnaire (SDQ) |
| Child-reported SDQ - Emotional score |  | Strengths and Difficulties Questionnaire (SDQ) |
| Child-reported SDQ - Conduct score |  | Strengths and Difficulties Questionnaire (SDQ) |
| Child-reported SDQ - Hyperactivity/Inattention score |  | Strengths and Difficulties Questionnaire (SDQ) |
| Child-reported SDQ - Peer relationship problems score |  | Strengths and Difficulties Questionnaire (SDQ) |
| Child-reported SDQ - Pro-social behaviour score |  | Strengths and Difficulties Questionnaire (SDQ) |
| Parent-reported SDQ - Impact score |  | Strengths and Difficulties Questionnaire (SDQ) |
| Parent-reported SDQ - Emotional score |  | Strengths and Difficulties Questionnaire (SDQ) |
| Parent-reported SDQ - Conduct score |  | Strengths and Difficulties Questionnaire (SDQ) |
| Parent-reported SDQ - Hyperactivity/Inattention score |  | Strengths and Difficulties Questionnaire (SDQ) |
| Parent-reported SDQ - Peer relationship problems score |  | Strengths and Difficulties Questionnaire (SDQ) |
| Parent-reported SDQ - Pro-social behaviour score |  | Strengths and Difficulties Questionnaire (SDQ) |
| Callous-unemotional traits | ICUT [2] is a 24-item questionnaire designed to provide a comprehensive assessment of callous- unemotional traits. ICUT was completed by both the young people and their parents/caregivers. | Inventory of Callous-Unemotional Traits (ICUT) |
| Parents' callous-unemotional traits |  | Inventory of Callous-Unemotional Traits (ICUT) |
| SRD - Volume of delinquency excluding violence towards siblings | The SRD allows for the assessment of the type and frequency of delinquent or law-breaking behaviour, self-reported by the young person. | Self-Report Delinquency measure (SRD) |
| SRD - Variety of delinquency excluding violence towards siblings |  | Self-Report Delinquency measure (SRD) |
| SRD - Volume of substance misuse |  | Self-Report Delinquency measure (SRD) |
| SRD - Variety of substance misuse |  | Self-Report Delinquency measure (SRD) |
| SRD - Peer illegal substance misuse scale |  | Self-Report Delinquency measure (SRD) |
| SRD - Peer delinquency scale |  | Self-Report Delinquency measure (SRD) |
| Onset of conduct problems (early or late) | Age at onset of antisocial behaviour: early (≤11 years) or late (>11 years). | Clinician decision at point of referral |
| Antisocial behaviour and attitudes | The ABAS [3] assesses antisocial cognitions in older children and adolescents. | Antisocial Beliefs and Attitudes Scales (ABAS) |
| Materialistic values | The Youth Materialism Scale [4] measures materialistic values in youth. | Youth Materialism Scale |
| **Mental health and wellbeing** |  |  |
| ADHD symptoms | Conners Rating Scale [5] provides a well-validated assessment of ADHD and learning vulnerabilities in  young people up to 18 years of age. | Conners Rating Scale (ADHD subscale) |
| Learning vulnerabilities |  | Conners Rating Scale (Learning & Language subscales) |
| Well-being and depression symptoms | The SMFQ [6] is a brief self-reported measure of childhood and adolescent depression focused on core depressive symptomatology. | Short Mood and Feelings Questionnaire (SMF) |
| Parental mental health | The GHQ [7] is a brief assessment of parental mental health, commonly used for the identification of mental health problems. | General Health Questionnaire (GHQ) |
| **Parenting assessment** |  |  |
| APQ - Positive parent score | The APQ [8] measures five dimensions of parenting. The measures used in this study was completed by the parent. | Alabama Parenting Questionnaire (APQ) |
| APQ - Parent involvement score |  | Alabama Parenting Questionnaire (APQ) |
| APQ - Monitoring/Supervision score |  | Alabama Parenting Questionnaire (APQ) |
| APQ - Corporal punishment score |  | Alabama Parenting Questionnaire (APQ) |
| APQ - Inconsistent discipline score |  | Alabama Parenting Questionnaire (APQ) |
| Family functioning (parental supervision and involvement) | The Loeber Caregiver Questionnaire [9] is a parent-completed questionnaire. We included the parental monitoring and supervision subscale in the present study. | Loeber Caregiver Questionnaire |
| **Family functioning** |  |  |
| FACES - Cohesion dimension score | The FACES-IV [10] was developed to evaluate the adaptability and cohesion dimensions in family interactions. This is the degree to which families have clear roles, responsibilities and boundaries within the family, as well as the degree to which family members feel close to and involved in each other’s lives. Completed by the parent/caregiver. | Family Adaptability and Cohesion Evaluation Scales (FACES-IV) |
| FACES - Flexibility dimension score |  | Family Adaptability and Cohesion Evaluation Scales (FACES-IV) |
| FACES - Family communication scale |  | Family Adaptability and Cohesion Evaluation Scales (FACES-IV) |
| FACES - Family satisfaction scale |  | Family Adaptability and Cohesion Evaluation Scales (FACES-IV) |
| Degree of conflict in the parental relationship | The CTS2 [11] is a measure that explores intra-family conflict and violence focusing particularly on intimate partner violence. Completed by the parent/caregiver. | Couple Conflicts Tactics Scale (CTS2) |
| Levels of expressed emotions | The LEE [12] is designed to measure to what degree the adolescent perceives lack of emotional support, intrusiveness and criticism in their major relationships within the family. Completed by the young person. | Levels of Expressed Emotions (LEE) |
| **Educational participation** |  |  |
| Registered in mainstream education | CA-SUS is a questionnaire developed specifically for the trial, designed to record all contact with health, social care, and criminal justice services. Completed by the parent/caregiver and young person. | Child and adolescent service use schedule (CA-SUS) |
| Registered in specialist education |  | Child and adolescent service use schedule (CA-SUS) |
| Statement of special educational needs |  | Child and adolescent service use schedule (CA-SUS) |
| Number of total school exclusions | Information on young people’s absences and expulsion was collected from the UK National Pupil Database. | National pupil database |
| Total number of days excluded |  | National pupil database |
| Proportion of unauthorised absences |  | National pupil database |

# Further information on machine learning algorithms

| **Machine learning algorithm** | **Description** |
| --- | --- |
| Multivariable logistic regression | - Extends univariable logistic regression to handle multiple predictor variables. For a binary classification task, the model predicts the probability of an outcome using a linear combination of predictors, or variables, as input. Each predictor is associated with a weight coefficient, and there is a final intercept term. The model learns by maximising the likelihood of the observed data during training. - Simple and interpretable, but susceptible to overfitting when there is high correlation between features (multicollinearity). |
| Elastic net regression [13] | - Seeks to reduce the risk of overfitting by penalising collinear feature variables. Balances the strengths of both Ridge and LASSO regression [14, 15]**.** Able to reduce the impact of less important features and also select a subset of the most important ones. - Particularly useful when dealing with datasets with multicollinearity, but may not be suitable for non-linear associations, and regression coefficients not easily interpretable. |
| Random forest [16] | - Constructs multiple decision trees [17] and ensembles the predictions by averaging or majority voting. Each tree is built on a random subset of the data and a random subset of features. Following training, each tree outputs a predicted class for classification, and the final result is determined by majority vote. - Does not assume a linear relationship, and works well with high-dimensional data. Risks overfitting if each tree is too deep, i.e. many decision boundaries per tree. Not easily interpretable. |
| Gradient boosting machine [18] | - Builds an ensemble of decision trees (or another weak learner), with each tree improving upon the predictions of the previous generation of trees. Trees are optimised to minimise a specified loss function (e.g. log loss) during training. Uses gradient descent to find the optimal parameters and build decision trees in a way that focuses on the most challenging examples in the training data. Subsampling (randomly sampling a subset of the training data to grow each decision tree) is one method to introduce randomness to the training process and reduce the risk of overfitting. The final prediction is the weighted sum of the predictions made by individual trees. - Able to handle complex relationships and achieve high accuracy, but requires careful hyperparameter tuning to prevent overfitting. Not easily interpretable. |

# Hyperparameter optimisation using grid search

Table C. Hyperparameters tested via 10-fold cross-validation and selected for building machine learning models

| **Model (Python class)** | **Hyperparameter^a^** | **Values tested via grid search** | **Metric used for model training** |  |  |
| --- | --- | --- | --- | --- | --- |
| Elastic net regression  (LogisticRegression^b^;  penalty=’elasticnet’,  solver=’saga’,  max_iter=5000) | l1_ratio | 0, 0.1, 0.2, 0.3, 0.4, 0.5, 0.6, 0.7, 0.8, 0.9, 1.0 | Accuracy |  |  |
|  | C | 0.001, 0.01, 0.1, 0.5, 1, 10, 100 |  |  |  |
|  |  |  |  |  |  |
| Random forest | criterion | Entropy | Accuracy |  |  |
| (ExtraTreesClassifier^c^) | max_features | 0.01,0.02,0.03,0.05,0.07,0.09,0.1 |  |  |  |
|  | max_depth | 3, 5, 7, 9, 11 |  |  |  |
|  | n_estimators | 100, 200, 300, 400, 500 |  |  |  |
|  |  |  |  |  |  |
| Gradient boosting machine  (XGBClassifier^d^) | n_estimators | 50, 100, 300, 500 | Logloss |  |  |
|  | learning_rate | 0.01, 0.03, 0.1 |  |  |  |
|  | subsample | 0.5, 0.75, 1.0 |  |  |  |
|  | max_depth | 1, 4, 6, 8, 10 |  |  |  |
| ^a^ If default values were used, they were not listed.  ^b^ See: [sklearn.linear_model.LogisticRegression — scikit-learn 1.3.1 documentation](https://scikit-learn.org/stable/modules/generated/sklearn.linear_model.LogisticRegression.html#sklearn.linear_model.LogisticRegression)  ^c^ See: [sklearn.ensemble.ExtraTreesClassifier — scikit-learn 1.3.1 documentation](https://scikit-learn.org/stable/modules/generated/sklearn.ensemble.ExtraTreesClassifier.html#sklearn.ensemble.ExtraTreesClassifier.predict)  ^d^ See: [Python API Reference — xgboost 2.1.0-dev documentation](https://xgboost.readthedocs.io/en/latest/python/python_api.html#module-xgboost.sklearn) | | |  |  |  |

# Top five features in machine learning-based prediction models

Average feature importance values and 95% CIs were calculated across the 100 random test sets. The top five features per model are displayed.

| **Feature** | **Mean (\|SHAP value\|)** | **95% Confidence Interval** | |
| --- | --- | --- | --- |
| **Multivariable logistic regression** |  |  | |
| Offender on referral | 0.36 | 0.35 | 0.36 |
| Site of clinical trial | 0.14 | 0.13 | 0.14 |
| Socioeconomic status | 0.10 | 0.09 | 0.11 |
| Source of referral | 0.08 | 0.07 | 0.08 |
| Gender | 0.05 | 0.04 | 0.05 |
| **+ additional predictors** |  |  |  |
| Offender on referral | 0.28 | 0.27 | 0.28 |
| SRD - Volume of substance misuse | 0.09 | 0.09 | 0.10 |
| Site of clinical trial | 0.09 | 0.08 | 0.09 |
| SRD - Variety of substance misuse | 0.09 | 0.08 | 0.09 |
| Source of referral | 0.06 | 0.06 | 0.06 |
| **Elastic net regression** |  |  |  |
| Offender on referral | 0.33 | 0.32 | 0.33 |
| Total number of offences | 0.08 | 0.08 | 0.09 |
| IQ | 0.08 | 0.07 | 0.08 |
| DAWBA conduct disorder diagnosis | 0.07 | 0.06 | 0.07 |
| Source of referral | 0.05 | 0.05 | 0.06 |
| **+ additional predictors** |  |  |  |
| Total number of offences | 0.12 | 0.12 | 0.12 |
| Offender on referral | 0.10 | 0.10 | 0.11 |
| Child-reported SDQ - Emotional score | 0.10 | 0.09 | 0.10 |
| SRD - Volume of delinquency excluding violence towards siblings | 0.10 | 0.09 | 0.10 |
| Antisocial Beliefs and Attitudes Scale | 0.06 | 0.06 | 0.07 |
| **Random forest** |  |  |  |
| Offender on referral | 0.16 | 0.15 | 0.16 |
| Site of clinical trial | 0.03 | 0.03 | 0.03 |
| DAWBA conduct disorder diagnosis | 0.03 | 0.03 | 0.03 |
| Gender | 0.02 | 0.02 | 0.02 |
| Source of referral | 0.02 | 0.02 | 0.02 |
| **+ additional predictors** |  |  |  |
| Offender on referral | 0.15 | 0.15 | 0.16 |
| DAWBA conduct disorder diagnosis | 0.02 | 0.02 | 0.02 |
| Site of clinical trial | 0.01 | 0.01 | 0.02 |
| Gender | 0.01 | 0.01 | 0.01 |
| Registered in specialist education | 0.01 | 0.01 | 0.01 |
| SRD= Self-Report Delinquency measure; DAWBA= Development and Well-being Assessment | | | |

# Completed TRIPOD Checklist

| **Section/Topic** | **Item** | **Checklist Item** | **Page** |
| --- | --- | --- | --- |
| **Title and abstract** | | | |
| Title | 1 | Identify the study as developing and/or validating a multivariable prediction model, the target population, and the outcome to be predicted. | 1 |
| Abstract | 2 | Provide a summary of objectives, study design, setting, participants, sample size, predictors, outcome, statistical analysis, results, and conclusions. | 1 |
| **Introduction** | | | |
| Background and objectives | 3a | Explain the medical context (including whether diagnostic or prognostic) and rationale for developing or validating the multivariable prediction model, including references to existing models. | 2 |
|  | 3b | Specify the objectives, including whether the study describes the development or validation of the model or both. | 2 |
| **Methods** | | | |
| Source of data | 4a | Describe the study design or source of data (e.g., randomized trial, cohort, or registry data), separately for the development and validation data sets, if applicable. | 2-3 |
|  | 4b | Specify the key study dates, including start of accrual; end of accrual; and, if applicable, end of follow-up. | 2-3 |
| Participants | 5a | Specify key elements of the study setting (e.g., primary care, secondary care, general population) including number and location of centres. | 2-3 |
|  | 5b | Describe eligibility criteria for participants. | 2-3 |
|  | 5c | Give details of treatments received, if relevant. | 2-3 |
| Outcome | 6a | Clearly define the outcome that is predicted by the prediction model, including how and when assessed. | 3 |
|  | 6b | Report any actions to blind assessment of the outcome to be predicted. | N/A |
| Predictors | 7a | Clearly define all predictors used in developing or validating the multivariable prediction model, including how and when they were measured. | 3-5; Supplement A |
|  | 7b | Report any actions to blind assessment of predictors for the outcome and other predictors. | N/A |
| Sample size | 8 | Explain how the study size was arrived at. | 2-3 |
| Missing data | 9 | Describe how missing data were handled (e.g., complete-case analysis, single imputation, multiple imputation) with details of any imputation method. | 5 |
| Statistical analysis methods | 10a | Describe how predictors were handled in the analyses. | 3-5 |
|  | 10b | Specify type of model, all model-building procedures (including any predictor selection), and method for internal validation. | 3, 5 |
|  | 10d | Specify all measures used to assess model performance and, if relevant, to compare multiple models. | 5 |
| Risk groups | 11 | Provide details on how risk groups were created, if done. | N/A |
| **Results** | | | |
| Participants | 13a | Describe the flow of participants through the study, including the number of participants with and without the outcome and, if applicable, a summary of the follow-up time. A diagram may be helpful. | 5 |
|  | 13b | Describe the characteristics of the participants (basic demographics, clinical features, available predictors), including the number of participants with missing data for predictors and outcome. | 5; Supplement A |
| Model development | 14a | Specify the number of participants and outcome events in each analysis. | 5 |
|  | 14b | If done, report the unadjusted association between each candidate predictor and outcome. | N/A |
| Model specification | 15a | Present the full prediction model to allow predictions for individuals (i.e., all regression coefficients, and model intercept or baseline survival at a given time point). | N/A* |
|  | 15b | Explain how to the use the prediction model. | 3 |
| Model performance | 16 | Report performance measures (with CIs) for the prediction model. | 6 |
| **Discussion** | | | |
| Limitations | 18 | Discuss any limitations of the study (such as nonrepresentative sample, few events per predictor, missing data). | 8 |
| Interpretation | 19b | Give an overall interpretation of the results, considering objectives, limitations, and results from similar studies, and other relevant evidence. | 7 |
| Implications | 20 | Discuss the potential clinical use of the model and implications for future research. | 8 |
| **Other information** | | | |
| Supplementary information | 21 | Provide information about the availability of supplementary resources, such as study protocol, Web calculator, and data sets. | 8 |
| Funding | 22 | Give the source of funding and the role of the funders for the present study. | 8 |
| * Complex machine learning models including gradient boosting machines do not have easily reportable model structures such as regression coefficients. We have instead reported model-agnostic feature importance values which give an indication of the operational basis of these models (Table 4 & Supplement D). | | | |

**References**

1. Goodman R, Scott S. Comparing the Strengths and Difficulties Questionnaire and the Child Behavior Checklist: is small beautiful? J Abnorm Child Psychol. 1999;27(1):17-24. doi:10.1023/a:1022658222914.

2. Essau CA, Sasagawa S, Frick PJ. Callous-unemotional traits in a community sample of adolescents. Assessment. 2006;13(4):454-69. doi:10.1177/1073191106287354.

3. Butler SM, Leschied AW, Fearon P. Antisocial Beliefs and Attitudes in Pre-adolescent and Adolescent Youth: the Development of the Antisocial Beliefs and Attitudes Scales (ABAS). Journal of Youth and Adolescence. 2007;36(8):1058-71. doi:10.1007/s10964-007-9178-2.

4. Goldberg ME, Gorn GJ, Peracchio LA, Bamossy G. Understanding materialism among youth. Journal of Consumer Psychology. 2003;13(3):278-88. doi:10.1207/NO_DOI.

5. Conners CK, Sitarenios G, Parker JD, Epstein JN. The revised Conners' Parent Rating Scale (CPRS-R): factor structure, reliability, and criterion validity. J Abnorm Child Psychol. 1998;26(4):257-68. doi:10.1023/a:1022602400621.

6. Sharp C, Goodyer IM, Croudace TJ. The Short Mood and Feelings Questionnaire (SMFQ): a unidimensional item response theory and categorical data factor analysis of self-report ratings from a community sample of 7-through 11-year-old children. J Abnorm Child Psychol. 2006;34(3):379-91. doi:10.1007/s10802-006-9027-x.

7. Goldberg DP, Gater R, Sartorius N, Ustun TB, Piccinelli M, Gureje O, et al. The validity of two versions of the GHQ in the WHO study of mental illness in general health care. Psychol Med. 1997;27(1):191-7. doi:10.1017/s0033291796004242.

8. Essau CA, Sasagawa S, Frick PJ. Psychometric Properties of the Alabama Parenting Questionnaire. Journal of Child and Family Studies. 2006;15(5):597-616. doi:10.1007/s10826-006-9036-y.

9. Loeber R, Farrington DP. Serious & violent juvenile offenders: Risk factors and successful interventions. Loeber R, Farrington DP, editors. Thousand Oaks, CA, US: Sage Publications, Inc; 1998. xxv, 507-xxv, p.

10. Olson D. FACES IV and the Circumplex Model: validation study. (1752-0606 (Electronic)).

11. Straus MA, Hamby SL, Boney-McCoy SUE, Sugarman DB. The Revised Conflict Tactics Scales (CTS2): Development and Preliminary Psychometric Data. Journal of Family Issues. 1996;17(3):283-316. doi:10.1177/019251396017003001.

12. Gerlsma C, Hale WW, 3rd. Predictive power and construct validity of the Level of Expressed Emotion (LEE) scale. Depressed out-patients and couples from the general community. (0007-1250 (Print)).

13. Zou H, Hastie T. Regularization and Variable Selection Via the Elastic Net. Journal of the Royal Statistical Society Series B: Statistical Methodology. 2005;67(2):301-20. doi:10.1111/j.1467-9868.2005.00503.x.

14. Tibshirani R. Regression Shrinkage and Selection via the Lasso. Journal of the Royal Statistical Society Series B (Methodological). 1996;58(1):267-88.

15. Hoerl AE, Kennard RW. Ridge Regression: Biased Estimation for Nonorthogonal Problems. Technometrics. 1970;12(1):55-67. doi:10.1080/00401706.1970.10488634.

16. Breiman L. Random Forests. Machine Learning. 2001;45(1):5-32. doi:10.1023/A:1010933404324.

17. Murthy SK. Automatic Construction of Decision Trees from Data: A Multi-Disciplinary Survey. Data Mining and Knowledge Discovery. 1998;2(4):345-89. doi:10.1023/A:1009744630224.

18. Friedman JH. Stochastic gradient boosting. Computational Statistics & Data Analysis. 2002;38(4):367-78. doi:<https://doi.org/10.1016/S0167-9473(01)00065-2>.
